# Knowledge mapping and global trends in the field of low-intensity pulsed ultrasound and endocrine and metabolic diseases: a bibliometric and visual analysis from 2012 to 2022

**DOI:** 10.3389/fendo.2023.1237864

**Published:** 2023-09-05

**Authors:** Guangdi Chu, Haitao Niu

**Affiliations:** Department of Urology, The Affiliated Hospital of Qingdao University, Qingdao, China

**Keywords:** low-intensity pulsed ultrasound, bibliometrics, visualization, endocrine and metabolic diseases, global trend

## Abstract

**Background:**

Low-intensity pulsed ultrasound (LIPUS) is a highly promising therapeutic method that has been widely used in rehabilitation, orthopedics, dentistry, urology, gynecology, and other multidisciplinary disease diagnoses and treatments. It has attracted extensive attention worldwide. However, there is currently a lack of comprehensive and systematic research on the current status and future development direction of the LIPUS field. Therefore, this study comprehensively analyzed LIPUS-related reports from the past decade using bibliometrics methods, and further conducted research specifically focusing on its application in endocrine and metabolic diseases.

**Methods:**

We downloaded LIPUS literature from 2012 to 2022 reported in the Web of Science Core Collection Science Citation Index-Expanded and Social Sciences Citation Index, and used bibliometric analysis software such as VOSviewer and CiteSpace to execute the analysis and visualize the results.

**Results:**

We searched for 655 English articles published on LIPUS from 2012 to 2022. China had the highest number of published articles and collaborations between China and the United States were the closest in this field. Chongqing Medical University was the institution with the highest output, and ULTRASOUND IN MEDICINE AND BIOLOGY was the journal with the most related publications. In recent years, research on the molecular mechanisms of LIPUS has continued to deepen, and its clinical applications have also continued to expand. The application of LIPUS in major diseases such as oxidative stress, regeneration mechanism, and cancer is considered to be a future research direction, especially in the field of endocrinology and metabolism, where it has broad application value.

**Conclusion:**

Global research on LIPUS is expected to continue to increase, and future research will focus on its mechanisms of action and clinical applications. This study comprehensively summarizes the current development status and global trends in the field of LIPUS, and its research progress in the field of endocrine and metabolic diseases, providing valuable reference for future research in this field.

## 1 Introduction

Low-intensity pulsed ultrasound (LIPUS) is a form of ultrasound that outputs in a pulsed wave mode, with energy intensity significantly lower than traditional ultrasound (1). LIPUS is considered a promising technology and has been widely applied in various clinical treatments and basic research (2). Its low frequency, low energy, and non-thermal effects have attracted the attention of numerous scholars (3). Increasing evidence shows that LIPUS can effectively stimulate osteoblasts, promote bone formation, and activate other stem/progenitor cells (4). LIPUS can enhance the vitality, proliferation, and multi-directional differentiation of various postnatal mesenchymal stem cells (MSCs) (2). Moreover, it can reduce the expression of pro-inflammatory cytokines, limit the infiltration of inflammatory cells, and regulate the phenotype of inflammatory cells (5). These beneficial changes may be related to increased blood flow, activated mitochondrial biogenesis, and anti-oxidative stress to promote muscle healing (6–8). Currently, LIPUS has been extensively used in tissue repair and regeneration, fracture healing, and inhibition of inflammatory responses. Additionally, it has significant potential therapeutic value in the field of urology, including chronic prostatitis/chronic pelvic pain syndrome (CP/CPPS) (9), erectile dysfunction (ED) (10, 11), stress urinary incontinence (SUI) (12), as well as gynecology, including premature ovarian insufficiency (13) and incomplete uterine involution (14).

Bibliometrics is a method of literature analysis that utilizes quantitative and qualitative perspectives to quantify and visualize published literature, thereby facilitating the evaluation of intrinsic connections and distribution patterns within research literature (15, 16). It offers a comprehensive and systematic assessment of the present research status, while also identifying potential avenues for future research within targeted fields. Bibliometrics effectively integrates information throughout the research process, enhances understanding, and assists in the deepening of knowledge regarding specific research areas (17, 18). It has been noted that there is currently a lack of bibliometric analysis focusing on the recent cooperation and research trends of LIPUS, with a focus on endocrinology and metabolism. Therefore, we conducted a systematic search of LIPUS-related literature in the Web of Science Core Collection (WoSCC) database and performed a comprehensive bibliometric analysis to clarify its current research status. We also combined with the latest published literature to analyze the research hotspots of LIPUS in the field of endocrinology and metabolic diseases to gain insights into future research directions in this field.

## 2 Materials and methods

### 2.1 Literature source and search

We conducted a comprehensive search of literature from 2012 to 2022 using the Science Citation Index Expanded (SCI-EXPANDED) and Social Sciences Citation Index (SSCI) databases in the WoSCC. To minimize bias caused by frequent database updates, we completed data retrieval and analysis on the same day. We searched for relevant publications in WoSCC using the following search strategy: TS = (“LIPUS” OR “Low-Intensity Pulsed Ultrasounds” OR “Pulsed Ultrasound, Low-Intensity” OR “Ultrasound, Low-Intensity Pulsed” OR “Low Intensity Pulsed Ultrasound”).

### 2.2 Inclusion and exclusion criteria

We systematically searched for literature related to LIPUS, limiting the document type to articles and reviews and language to English. Conference abstracts and other article types were excluded, as well as articles in languages other than English. We also excluded any other articles that were not relevant to the research subject or had been retracted.

### 2.3 Literature analysis method

We downloaded the literature records in plain text format from WoSCC and used VOSviewer for analysis of countries, universities, authors, and keywords. We used CiteSpace for burst word analysis, with the time was set from January 2012 to December 2022, the type was set as “keywords,” and other parameters set to default values. Additionally, we used R software to calculate the annual number of articles published and plotted a line graph.

## 3 Results

### 3.1 Search results

**Figure 1:**
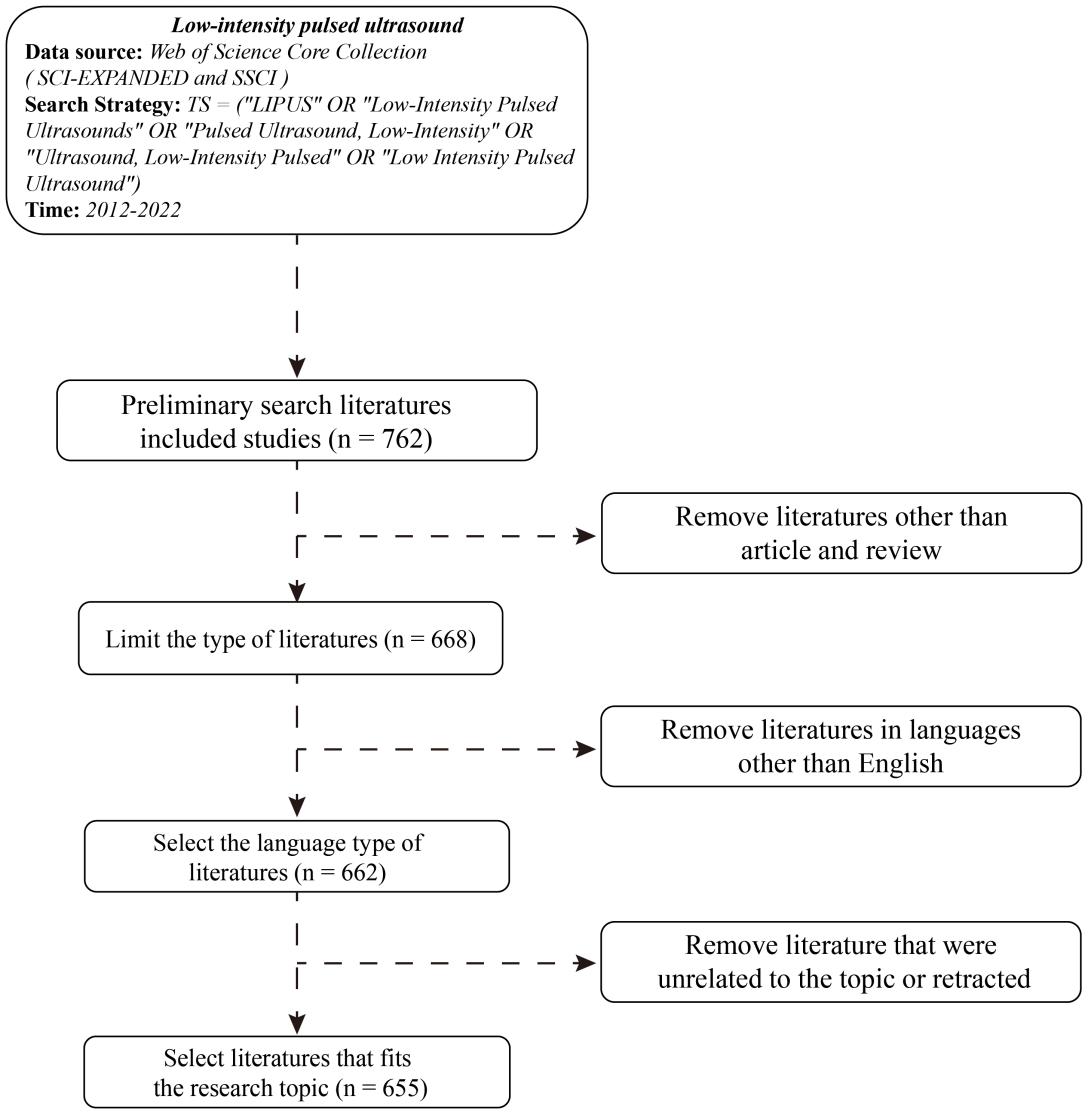
Literature search process and its inclusion and exclusion criteria.

The process of inclusion and exclusion of articles is shown in **Figure 1**. From January 1, 2012 to December 31, 2022, a total of 762 articles were obtained. By limiting the document type to article or review, 668 articles were obtained. Limiting the language type to English resulted in 662 articles. After removing literature that was unrelated to the topic or retracted, 655 articles were included for subsequent analysis.

### 3.2 Analysis of global trends

**Figure 2:**
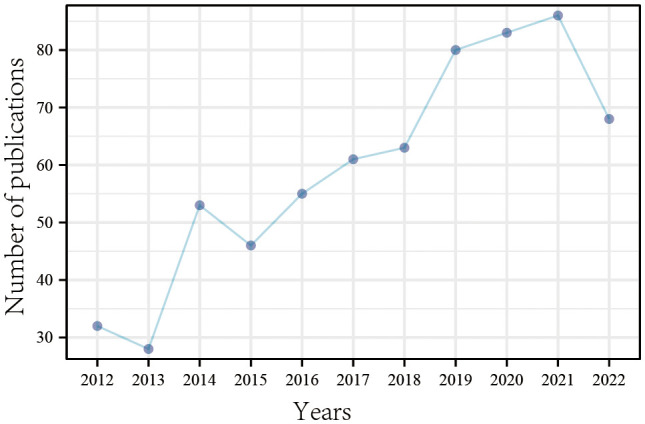
The number of publications per year from 2012 to 2022.

Through statistics of the annual publication volume (**Figure 2**), we found that the number of publications related to LIPUS research worldwide has shown a fluctuating upward trend. 2014 was a short-term peak period, and since 2015, the number of publications has been increasing year by year, especially in two periods from 2013-2014 and 2018-2019, where the publication speed increased rapidly, proving its rapid rise in global research popularity. In 2022, the publication volume showed a short-term decline, but the annual publication volume still exceeded 60 articles, which is at a relatively high level and consistent with the overall trend of fluctuating upward. In the past five years, the number of publications related to LIPUS research has remained at a high level, indicating that LIPUS is still a hot topic in current international research and continues to receive much attention.

### 3.3 Analysis of the contributions of countries and regions

**Figure 3:**
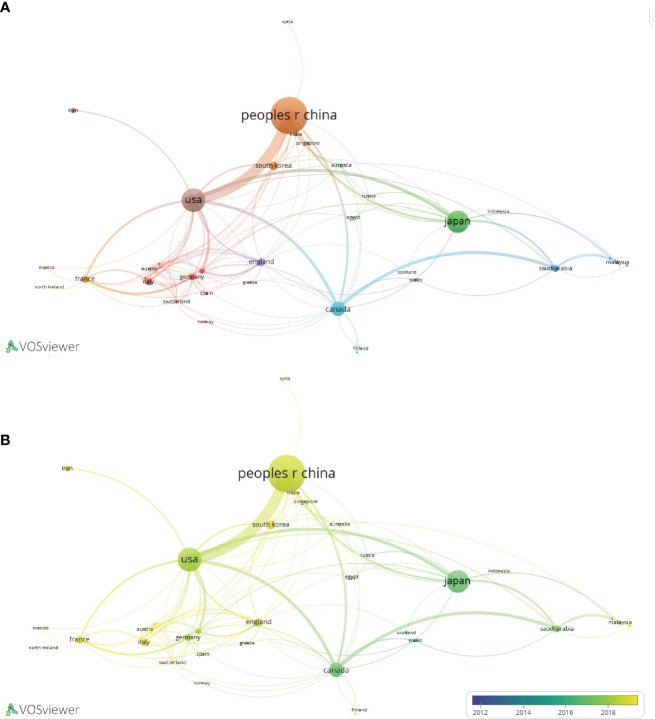
Collaboration network diagram of LIPUS literature publication countries. **(A)** Collaborative relationships in research in this field between different countries. **(B)** According to the time trend analysis of the relationship between cooperation among countries and years, the lighter the color represents the closer the year in which their cooperative relationship was developed.

A total of 46 countries have contributed to international LIPUS research, among which China, the United States, and Japan are the top three countries with the highest number of related article publications, accounting for 81.2% (532/655) (**Table 1**). The top three countries with the highest average citation frequency of published articles are the Netherlands, France, and Italy. Stable cooperation and exchanges have been formed among the 46 countries, especially between China and the United States (**Figure 3A**). By analyzing the changes in time distribution, we found that early LIPUS research was mainly concentrated in countries such as Japan and Canada. However, it gradually gained attention and developed rapidly in other countries around 2018 (**Figure 3B**).

**Table 1:** The top 20 countries with the most LIPUS publications from 2012 to 2022.

Country	Documents	Citations	Average citations
CHINA	295	4531	15.4
USA	125	2332	18.7
JAPAN	112	1555	13.9
CANADA	55	1024	18.6
SOUTH KOREA	25	211	8.4
ENGLAND	21	420	20.0
FRANCE	19	428	22.5
ITALY	18	383	21.3
GERMANY	17	325	19.1
SAUDI ARABIA	16	171	10.7
BRAZIL	15	138	9.2
NETHERLANDS	12	408	34.0
IRAN	12	93	7.8
TURKEY	11	88	8.0
INDIA	7	86	12.3
MALAYSIA	7	70	10.0
AUSTRALIA	6	95	15.8
WALES	5	74	14.8
SPAIN	5	36	7.2
SINGAPORE	5	33	6.6

### 3.4 Analysis of the contributions of universities and institutions

**Figure 4:**
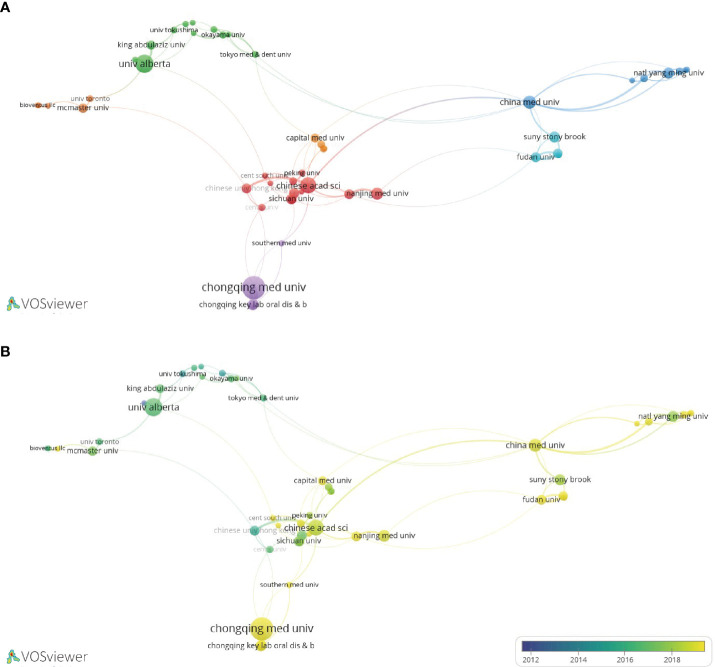
Collaboration network diagram of universities or institutions in LIPUS literature publication. **(A)** Collaborative relationships in research in this field among different universities or institutions. **(B)** According to the time trend analysis of the relationship between cooperation among universities or institutions and years, the lighter the color represents the closer the year in which their cooperative relationship was developed.

A total of 794 organizations have contributed to LIPUS research, among which Chongqing Medical University, University of Alberta, and Chinese Academy of Sciences are the top three organizations with the highest number of publications (**Table 2**). We selected the top 50 organizations in terms of publication volume for inter-organizational collaboration analysis and found that domestic Chinese organizations occupy the majority within the top 50 organizations, forming a relatively stable cooperative relationship (**Figure 4A**). Looking at the time trend, early LIPUS research was mainly carried out by related institutions in Japan and other countries. However, around 2018, research on LIPUS in China rose in popularity and produced many academic achievements (**Figure 4B**).

**Table 2:** The top 20 universities or institutions with the most LIPUS publications from 2012 to 2022.

Organization	Documents	Citations	Average citations
Chongqing medical university	52	698	13.4
University of Alberta	34	483	14.2
Chinese Academy of Sciences	28	514	18.4
China Medical University	19	299	15.7
Nanjing Medical University	17	300	17.6
National Yang-Ming University	15	511	34.1
Stony Brook University	15	251	16.7
Chongqing Key Laboratory of Oral Diseases and Biomedical Sciences	15	236	15.7
SiChuan University	14	242	17.3
The Chinese University of Hong Kong	13	416	32.0
Fudan University	13	107	8.2
Municipal Key Laboratory of Oral Biomedical Engineering of Chongqing Higher Education Institutions	12	149	12.4
Zhejiang University	12	124	10.3
Nanjing University	12	91	7.6
McMaster University	11	244	22.2
Shaanxi Normal University	11	105	9.5
Tohoku University	10	219	21.9
Shanghai Jiao Tong University	10	163	16.3
King abdulaziz university	10	100	10.0
Capital Medical University	10	82	8.2

### 3.5 Analysis of the contributions of authors

**Figure 5:**
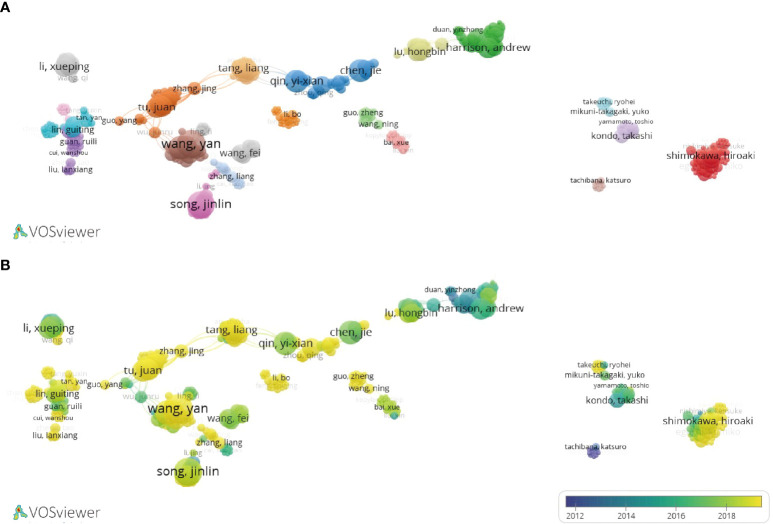
Collaboration network diagram of scholars in LIPUS literature publication. **(A)** Collaborative relationships in research in this field among different scholars. **(B)** According to the time trend analysis of the relationship between cooperation among scholars and years, the lighter the color represents the closer the year in which their cooperative relationship was developed.

A total of 2,973 authors have contributed to publications in this field, among which Tarek El-Bialy, Yan Wang, and Feng-Yi Yang are the top three authors with the highest number of publications (**Table 3**). Feng-Yi Yang is also the author with the highest number of citations. In terms of average citation frequency, Andrew Harrison, Kwok-Sui Leung, and Feng-Yi Yang are the top three scholars. Analyzing the collaborative relationship between authors, it can be found that international LIPUS research has formed a relatively stable collaborative group (**Figure 5**).

**Table 3:** The top 20 scholars with the most LIPUS publications from 2012 to 2022.

Author	Documents	Citations	Average citations
Tarek El-Bialy	21	259	12.3
Feng-Yi Yang	17	507	29.8
Yan Wang	17	219	12.9
Jinlin Song	16	299	18.7
Wenzhi Chen	13	136	10.5
Jie Chen	12	220	18.3
Yixian Qin	12	191	15.9
Andrew Harrison	10	360	36.0
Kwok-Sui Leung	10	327	32.7
Eiji Tanaka	10	195	19.5
Jie Li	10	161	16.1
Juan Tu	10	89	8.9
Liang Tang	10	83	8.3
Dong Zhang	10	82	8.2
Xueping Li	9	222	24.7
Jinyun Chen	9	95	10.6
Lijun Sun	9	83	9.2
Xiasheng Guo	9	81	9.0
Qiang Lin	8	219	27.4
Peng Xia	8	214	26.8

### 3.6 Analysis of highly influential journals

This study indicates that a total of 292 journals have published research on LIPUS. The results show that the journal with the highest output is ULTRASOUND IN MEDICINE AND BIOLOGY (n=60). It is followed by ULTRASONICS (n=21), SCIENTIFIC REPORTS (n=18) and JOURNAL OF ULTRASOUND IN MEDICINE (n=18) (**Table 4**). ULTRASOUND IN MEDICINE AND BIOLOGY is also the most cited journal, with a total of 744 citations. This indicates that these journals have strong academic influence in the field of LIPUS.

**Table 4:** The top 20 journals with the most LIPUS publications from 2012 to 2022.

Source	Documents	Citations	Average citations
ULTRASOUND IN MEDICINE AND BIOLOGY	60	744	12.4
ULTRASONICS	21	483	23.0
SCIENTIFIC REPORTS	18	494	27.4
JOURNAL OF ULTRASOUND IN MEDICINE	18	238	13.2
PLOS ONE	12	420	35.0
INTERNATIONAL JOURNAL OF MOLECULAR SCIENCES	12	136	11.3
ARCHIVES OF ORAL BIOLOGY	11	126	11.5
BMC MUSCULOSKELETAL DISORDERS	10	92	9.2
JOURNAL OF HARD TISSUE BIOLOGY	9	33	3.7
BIOMED RESEARCH INTERNATIONA	8	95	11.9
INJURY-INTERNATIONAL JOURNAL OF THE CARE OF THE INJURED	7	173	24.7
JOURNAL OF ORTHOPAEDIC RESEARCH	7	167	23.9
AMERICAN JOURNAL OF TRANSLATIONAL RESEARCH	7	85	12.1
IEEE TRANSACTIONS ON ULTRASONICS FERROELECTRICS AND FREQUENCY CONTROL	7	60	8.6
CEREBRAL CORTEX	6	138	23.0
BRAIN STIMULATION	5	222	44.4
JOURNAL OF ORTHOPAEDIC SCIENCE	5	167	33.4
STEM CELL RESEARCH 8 THERAPY	5	153	30.6
ANNALS OF BIOMEDICAL ENGINEERING	5	131	26.2
BONE	5	128	25.6

### 3.7 Analysis of co-citation networks

**Figure 6:**
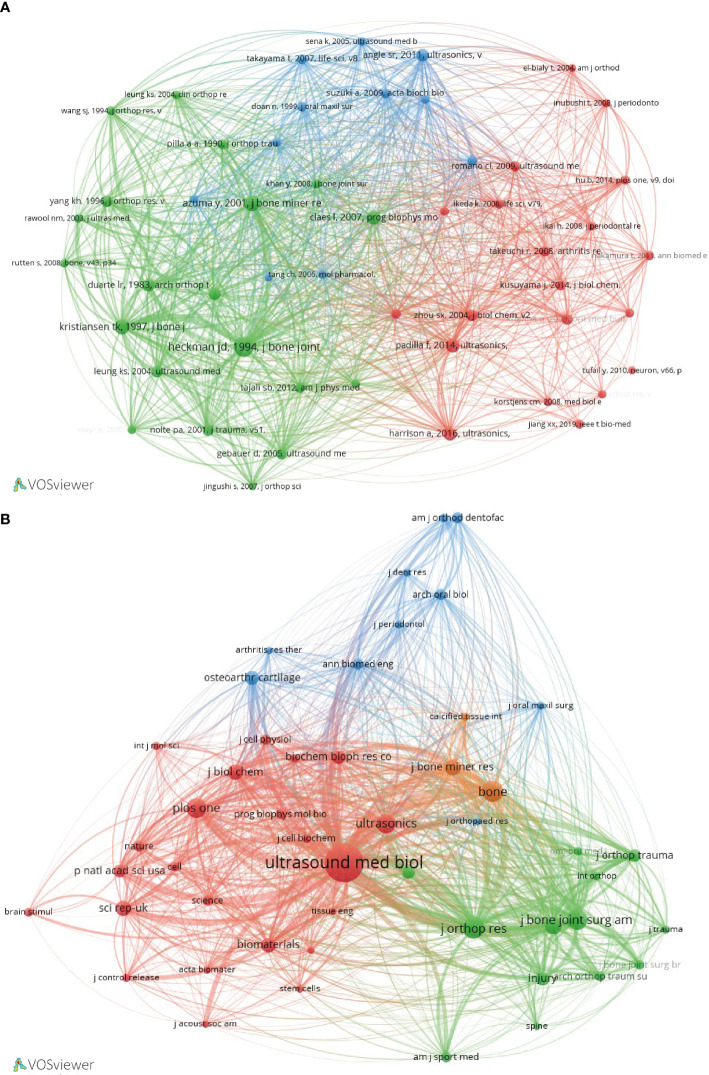
Co-citation network analysis of LIPUS literature. **(A)** Network cluster diagram of co-cited literature. **(B)** Cluster diagram of the main journals in which co-cited literatures were published.

We employed VOSviewer, a visualization software tool, to construct a co-citation network of references that encompasses 19,325 individual references across 3,832 distinct academic journals. Larger nodes represent references with higher citation frequencies, and thicker lines represent higher frequencies of co-citation. These co-cited articles can be divided into three main clusters (**Figure 6A**) and are mainly published in journals such as ULTRASOUND IN MEDICINE AND BIOLOGY, BONE, and JOURNAL OF ORTHOPAEDIC RESEARCH (**Figure 6B**).

### 3.8 Analysis of keywords

**Figure 7:**
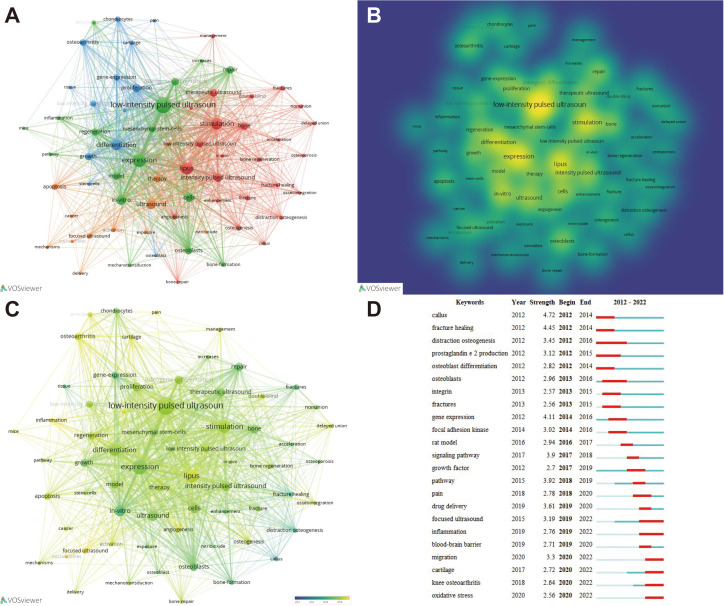
Analysis of keywords and burst word. **(A)** Cluster analysis of keywords and results. **(B)** Presentation of keyword occurrence frequency in the form of density plot. **(C)** According to the time trend analysis of the relationship between keywords and years, the lighter the color represents the closer the year in which the keyword appeared. **(D)** Identification of the most relevant keywords to LIPUS through burst words analysis.

From the retrieved records, a total of 3,004 keywords were extracted. We selected 67 keywords with a frequency of more than 15 occurrences, which can be divided into four clusters (**Figure 7A**). The top five keywords are low-intensity pulsed ultrasound (195 times), expression (141 times), LIPUS (125 times), stimulation (117 times), and differentiation (103 times), which encompass the most core keywords in each cluster (**Figure 7B**).

The identification of burst words, defined as keywords that exhibit a significant rise in frequency during a condensed time frame, is indicative of research hotspots within the respective field. Through an examination of time trends and burst words, one can gain insight into overarching research trends and developmental directions. Through temporal trend analysis and burst word analysis, we found that migration, cartilage, knee osteoarthritis, and oxidative stress related to LIPUS have become research hotspots since 2020 and have a continuous trend, and may continue to lead the direction of LIPUS research in the future (**Figures 7C, D**).

### 3.9 Analysis of the latest research hotspots on LIPUS in endocrine and metabolic diseases

**Figure 8:**
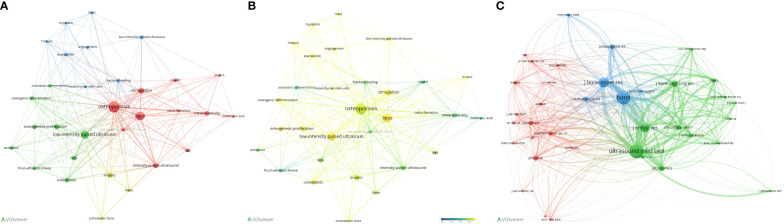
Analysis of keywords and co-citation network analysis of LIPUS in Endocrine and Metabolic Diseases. **(A)** Cluster analysis of keywords and results. **(B)** According to the time trend analysis of the relationship between keywords and years, the lighter the color represents the closer the year in which the keyword appeared. **(C)** Cluster diagram of the main journals in which co-cited literatures were published.

Currently, research on the application of LIPUS in endocrine and metabolic diseases is steadily increasing, but there is still a lack of systematic summarization. Therefore, we conducted further analysis on hypopituitarism, thyroid diseases, adrenal cortical diseases, pheochromocytoma, gout, osteoporosis, and other typical endocrine and metabolic diseases based on preliminary search results. Through further screening, we obtained a total of 326 relevant keywords, with 31 keywords appearing three or more times (**Figure 8A**). Among these keywords, “osteoporosis” had the highest frequency of occurrence. Time trend analysis reveals that osteoporosis serves as an early and mature example of LIPUS application, from basic research to clinical studies. And, in recent years, emerging areas such as stem cells and proliferation have become new research directions for LIPUS (**Figure 8B**).

The co-citation cluster analysis revealed that the selected articles cited a total of 1697 references, which were derived from 658 journals. Among these journals, 37 had been cited more than 10 times. ULTRASOUND IN MEDICINE AND BIOLOGY (n=105), BONE (n=105), JOURNAL OF BONE AND MINERAL RESEARCH (n=69), JOURNAL OF ORTHOPAEDIC RESEARCH (n=59), and JOURNAL OF BONE and JOINT SURGERY-AMERICAN VOLUME (n=40) were the top five most frequently cited journals, primarily focused on ultrasound and orthopedic fields (**Figure 8C**).

## 4 Discussion

In this global study, we conducted bibliometric analysis to summarize the collaboration network, research trends and hotspots, as well as future directions of LIPUS research. A total of 655 records were retrieved and published in 292 journals by 2973 authors from 794 institutions in 46 countries/regions. This is the first systematic study using bibliometric methods to analyze the development patterns of LIPUS over the past decade and analyze its research in the field of endocrine and metabolic diseases, which will guide its future development direction.

The results of this study indicate that China has the highest number of publications (n=295), followed by the United States (n=125), Japan (n=112), Canada (n=55), and South Korea (n=25), suggesting that LIPUS-related research is still relatively dominated by developed countries, which also reflects the correlation between scientific research, economic investment, and national social economy. The citation frequency of published articles can represent the academic influence of the results to a certain extent. Currently, China (n=4531), the US (n=2332), Japan (n=1555), and Canada (n=1024) have the highest total citation frequencies, consistent with the publication ranking. However, in terms of the average citation frequency of published articles, the top five countries are the Netherlands, France, Italy, the UK, and Germany, indicating that these countries may have higher academic influence in this field.

Regarding the distribution of patterns of institutions publishing relevant research and their national origins, the overall trend is consistent. In terms of the number of publications, four of the top five institutions are from China, namely Chongqing Medical University (n=52), Chinese Academy of Sciences (n=28), China Medical University (n=19), and Nanjing Medical University (n=17), while the other is the University of Alberta in Canada (n=34). Moreover, Chongqing Medical University has the highest total citation frequency of published articles (n=698) worldwide. Scholars planning to conduct LIPUS-related research should pay close attention to the academic achievements of this institution in the future.

In terms of author distribution, Tarek El-Bialy ranks first with 21 publications, focusing on the application of LIPUS in oral medicine-related areas such as the maxilla, mandible, alveolar bone, and orthodontics (19–24). Feng-Yi Yang and Yan Wang follow closely with 17 publications each. Feng-Yi Yang has the highest total citation frequency and primarily focuses on combining LIPUS to treat neurological disorders. He proved that LIPUS stimulation can be a potential therapeutic method by accelerating myelin regeneration through weakening glial cell activation, enhancing mature oligodendrocyte density, and increasing the production of BDNF (25). Abdominal LIPUS stimulation can combat neuroinflammation by enhancing tight junction protein levels and inhibiting colonic inflammation (26). LIPUS stimulation can serve as a potential therapeutic tool against Parkinson’s disease, possibly combined with its neural regenerative and protective effects (27, 28), and has therapeutic effects on erectile dysfunction caused by cavernous nerve injury (29). The scholar with the highest average citation frequency is Professor Andrew Harrison from The Chinese University of Hong Kong. His main academic contributions include exploring the effects of different therapeutic ultrasound intensities on rat fracture healing (30), demonstrating the positive effect of far-field LIPUS on stimulating bone cells and promoting mechanical transmission between bone and osteoblasts (31), summarizing the patterns and mechanisms of LIPUS in repairing fractures and conducting clinical trials of LIPUS in patients with non-healing chronic fractures (32–35). Scholars planning to study relevant research on LIPUS in orthopedics should pay attention to the research progress of Professor Andrew Harrison in the future.

LIPUS has significant clinical value in the treatment of various diseases and has been recognized by numerous high-level journals and editors. Nearly 300 journals have published reports related to LIPUS, with the top three journals in terms of number of published studies being ULTRASOUND IN MEDICINE AND BIOLOGY, ULTRASONICS, and SCIENTIFIC REPORTS, which also have the highest total citation frequencies. The top three journals in terms of average citation frequency are BRAIN STIMULATION, PLOS ONE, and JOURNAL OF ORTHOPAEDIC SCIENCE.

High-frequency keywords and burst words help scholars clarify future research directions in related fields. After keyword network and burst word analysis, we found that LIPUS-related research has gradually progressed from previous growth factors (36, 37), fracture healing (38–41), and integrins (42–44) to fields such as oxidative stress (45), regeneration (46, 47), and tumors (48–50). This suggests that the exploration of molecular biological mechanisms for LIPUS is gradually deepening, and its application areas are gradually expanding to major diseases such as cancer, proving once again the broad application prospects and clinical value of LIPUS.

The value of LIPUS in endocrine and metabolic diseases has attracted increasing attention. Han et al. found that LIPUS could increase testosterone levels in animal models and alleviate Leydig cell aging *in vitro (* 51). Furthermore, combining LIPUS with tadalafil effectively treats erectile dysfunction in patients with type 2 diabetes (52), and promotes nerve regeneration and improves erectile function by enhancing Schwann cell proliferation, migration, and expression of neurotrophic factors such as nerve growth factor (53). In terms of bone metabolism, Wu et al. have demonstrated that LIPUS can accelerate osteoblast differentiation and promote bone formation in a rat model of osteoporosis (54), and its ultrasound intensity is closely related to its therapeutic effect on osteoporosis (55, 56). Diabetes is a typical disease caused by glucose metabolism disorder, with a long course and many complications. LIPUS has unique effects in regulating glucose metabolism, and has been extensively studied. It has been shown to prevent muscle atrophy in rats with type 1 diabetes (57), and lower blood pressure in hypertensive patients with type 2 diabetes (58). LIPUS shows potential as a new therapy for type 2 diabetes (59). Our study stands at the forefront of summarizing the role and mechanisms of LIPUS from the perspective of endocrine and metabolic diseases, which will facilitate further research in this field by scholars in the future. However, our search has also revealed that, although research on LIPUS in this field is gradually gaining momentum, there is still significant room for further exploration, particularly in relation to specific diseases. The search results indicate that the most well-established application of LIPUS is in the study of osteoporosis. It has progressed from preliminary research on cellular and molecular mechanisms to practical clinical applications, and has shown promising therapeutic effects. There are also relevant studies on diabetes-related osteoporosis, muscle atrophy, erectile dysfunction, among others. However, research on specific diseases such as thyroid disorders and gout remains limited, with most studies focusing on the cellular and molecular levels, still some distance away from clinical application. However, it is well known that many endocrine and metabolic diseases are influenced by hormonal actions or metabolic disruptions. The specific regulatory mechanisms of LIPUS on substances such as glucose, lipids, hormones, and bone have been extensively documented, providing a foundation for its future clinical applications and driving advancements in the field.

Undoubtedly, this study, like other bibliometric studies, has certain limitations. Firstly, the data source mainly comes from the core dataset of WoSCC and lacks other databases, which may result in some omissions in the data we included. Secondly, the language type set for this study is only limited to English, which may cause relevant research in other languages to be missed. In addition, some newly published research results may be missed due to lack of inclusion in the database. Therefore, in the future, we will continue to update the data, pay attention to other non-English literature reports, and the latest published research.

## 5 Conclusion

Through comprehensive bibliometric analysis, we systematically analyzed global research on LIPUS over the past decade. Overall, the number of publications showed a fluctuating upward trend and the annual publication volume has now stabilized at a high level. In recent years, China and the United States have made the greatest contributions to LIPUS research, and their cooperation and exchanges have been the closest. The research interest in LIPUS for endocrine and metabolic diseases is steadily increasing, with a particular focus on areas such as regulation of glucose metabolism, lipid metabolism, and bone metabolism. Furthermore, LIPUS has shown promising applications in various diseases, with osteoporosis serving as a notable example. Based on the current development trend, it is expected that research on LIPUS will continue to increase globally, and exploring the related molecular mechanisms of LIPUS and expanding its clinical applications will be the future direction of research, especially in the field of endocrine and metabolic diseases.

## Data availability statement

The original contributions presented in the study are included in the article/supplementary material. Further inquiries can be directed to the corresponding author.

## Author contributions

GC designed the whole study, analyzed the data and drafted the manuscript. HN revised the manuscript. All authors read and approved the final manuscript. All authors contributed to the article and approved the submitted version.
